# Differing alterations of sodium currents in small dorsal root ganglion neurons after ganglion compression and peripheral nerve injury

**DOI:** 10.1186/1744-8069-4-20

**Published:** 2008-05-30

**Authors:** Zhi-Jiang Huang, Xue-Jun Song

**Affiliations:** 1 Department of Neurobiology, Parker University Research Institute, Dallas, TX 75229, USA; 2 Jiangsu Province Key Laboratory of Anesthesiology and Center for Pain Research and Treatment, Xuzhou Medical College, Xuzhou, Jiangsu 221002, PRoC

## Abstract

Voltage-gated sodium channels play important roles in modulating dorsal root ganglion (DRG) neuron hyperexcitability and hyperalgesia after peripheral nerve injury or inflammation. We report that chronic compression of DRG (CCD) produces profound effect on tetrodotoxin-resistant (TTX-R) and tetrodotoxin-sensitive (TTX-S) sodium currents, which are different from that by chronic constriction injury (CCI) of the sciatic nerve in small DRG neurons. Whole cell patch-clamp recordings were obtained *in vitro *from L_4 _and/or L_5 _dissociated, small DRG neurons following *in vivo *DRG compression or nerve injury. The small DRG neurons were classified into slow and fast subtype neurons based on expression of the slow-inactivating TTX-R and fast-inactivating TTX-S Na^+ ^currents. CCD treatment significantly reduced TTX-R and TTX-S current densities in the slow and fast neurons, but CCI selectively reduced the TTX-R and TTX-S current densities in the slow neurons. Changes in half-maximal potential (V_1/2_) and curve slope (*k*) of steady-state inactivation of Na^+ ^currents were different in the slow and fast neurons after CCD and CCI treatment. The window current of TTX-R and TTX-S currents in fast neurons were enlarged by CCD and CCI, while only that of TTX-S currents in slow neurons was increased by CCI. The decay rate of TTX-S and both TTX-R and TTX-S currents in fast neurons were reduced by CCD and CCI, respectively. These findings provide a possible sodium channel mechanism underlying CCD-induced DRG neuron hyperexcitability and hyperalgesia and demonstrate a differential effect in the Na^+ ^currents of small DRG neurons after somata compression and peripheral nerve injury. This study also points to a complexity of hyperexcitability mechanisms contributing to CCD and CCI hyperexcitability in small DRG neurons.

## Background

Nerve injury produces dorsal root ganglion (DRG) neuron hyperexcitability, which is thought to underlie neuropathic pain by causing central sensitization. The voltage-gated sodium channels (VGSCs) can be dynamically regulated after axonal injury or peripheral inflammation and play important roles in modulating neural excitability [1,2]. The VGSCs are critically important for electrogenesis and nerve impulse conduction, and a target for important clinically relevant analgesics. However, mechanisms of the VGSCs contributing to hyperexcitability of DRG neurons and neuropathic pain remain unclear and the observations are controversial. For instance, inhibition or specific knock-down of tetrodotoxin-resistant (TTX-R) current Nav1.8 channel can effectively suppress neuropathic pain [3-5], while the Nav1.8 mRNA, protein and current are substantially decreased in DRG neurons in axotomized DRG neurons [6-9] or sciatic nerve injury [10]. The tetrodotoxin-sensitive (TTX-S) current Nav1.7 channel plays a critical role in various pain conditions [1], but nociceptors specific deletion of Nav1.7 did not eliminate neuropathic pain behavior in mice [11]. Thus, there is a need to further investigate roles of the VGSCs in different neuropathic pain conditions.

Different from nerve injury models that produce injury to the peripheral axons of DRG neurons such as the chronic constriction injury (CCI) of the sciatic nerve, chronic compression of DRG (CCD) is used as an animal model that produces injury directly to DRG somata. We have shown that CCD treatment produces behavioral hyperalgesia and allodynia and DRG neuron hyperexcitability in rats [12-14]. However, ionic mechanisms contributing to CCD-induced neural hyperexcitability remain unclear. A recent study shows that TTX-R Na^+ ^currents are upregulated in the cutaneous medium-sized CCD DRG neurons [15], which is somewhat different from the findings in axon injury models. The small DRG neurons most are nociceptive and play critical roles in neuropathic pain. Expression of the Na^+ ^currents is different between small- and medium-sized DRG neurons in CCI rats [16]. However, ionic mechanisms have not been investigated in these small neurons after CCD treatment. The purpose of this study was to analyze the effects of CCD on the properties of TTX-R and TTX-S Na^+ ^currents in the small DRG neurons. Because of the complex and diverse expression of the VGSCs in different neuropathic pain conditions, we compared alterations of density and kinetic property of the TTX-R and TTX-S Na^+ ^currents in CCD with CCI DRGs in the same recording condition. This study provides sodium channel mechanisms underlying CCD-induced DRG neuron hyperexcitability and behavioral hyperalgesia and indicates different effects of CCD- and CCI-treatment on the TTX-R and TTX-S Na^+ ^currents.

Preliminary data have been published in an abstract form [17].

## Results

### CCD and CCI produced thermal hyperalgesia

**Figure 1:**
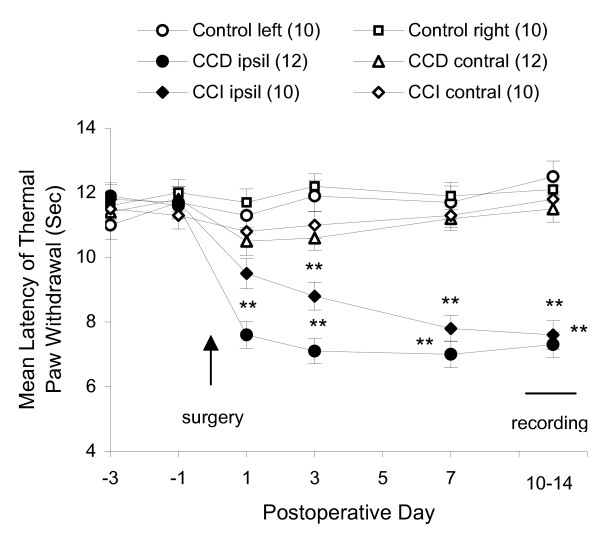
**Thermal hyperalgesia following CCD- or CCI-treatment in rats.** Repeated measurements are shown of thermal sensitivity of the foot withdrawal response in CCD-, CCI- and control rats. Numbers of rats used in each group are indicated in the parentheses. The arrow indicates the point of surgery of CCD or CCI performed. The dash line above "recording" indicates the period of time that the rats were sacrificed for electrophysiological recordings and the data were collected at different days of postoperative 10–14. **P < 0.01 indicate significant differences between groups of CCD ipsilateral or CCI ipsilateral to the other groups.

We began by confirming with earlier demonstrations that CCD- or CCI-treatment produced pain and hyperalgesia. All the CCD- and CCI-treated rats showed behavioral indications of thermal hyperalgesia. Withdrawal latencies of the foot ipsilateral to CCD or CCI treatment decreased significantly from the preoperative values. Withdrawal latencies of the foot contralateral to CCD or CCI treatment and both feet in control groups did not show significant change during the period of time. Data are shown in Fig. 1. All of these rats were used later for electrophysiological recordings during 10–14 postoperative days.

### TTX-R and TTX-S Na^+ ^currents in small neurons from CCD, CCI and control DRGs

Majority of the small DRG neurons express both TTX-R and TTX-S Na^+ ^currents, which have different activation and inactivation properties [6,8]. Prepulse inactivation, which takes advantage of differences in inactivation properties of the TTX-R and TTX-S currents, was used to separate the slow-inactivating TTX-R, and fast-inactivating TTX-S Na^+ ^currents [6,18]. A current-voltage protocol with a 700 ms prepulse to -120 mV followed by respective test pulse was applied first at a holding potential of -80 mV to obtain the total Na^+ ^current. The slow-inactivating, TTX-R currents were recorded using a prepulse of 700 ms -50 mV before the test pulse. This protocol inactivates the TTX-S currents while leaving the TTX-R current intact. TTX-S currents were then obtained by subtracting the TTX-R currents from the total Na^+ ^current in the cells. These protocols allowed simultaneous measurement of both TTX-R and TTX-S currents in each neuron recorded. In some neurons, the TTX-R currents were recorded with existence of TTX (300 nM, n = 6). The TTX-R and TTX-S currents were similar to that recorded by using protocols and the Na^+ ^currents were completely blocked by lidocaine (200 μM, n = 6) (data not shown). These data were similar to that described previously [6].

**Figure 2:**
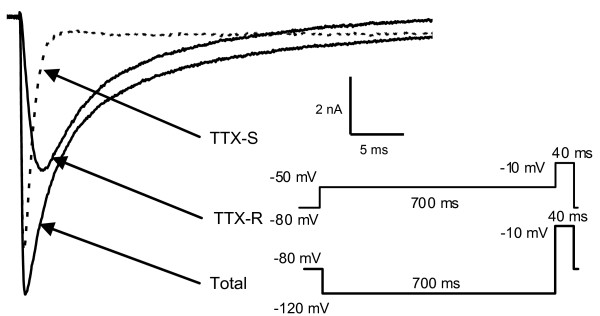
**Representative Na^+ ^currents traces recorded in a small neuron from a control DRG. **The total current was recorded with a 700 ms prepulse to -120 mV followed by the test pulse. The TTX-R Na^+ ^current was recorded with a 700 ms prepulse to -50 mV followed by a test pulse. The TTX-S Na^+ ^current was obtained by digital subtraction of the TTX-R current from the total current. All the test pulses were 40 ms to -10 mV pulses. The holding potential was at -80 mV.

One hundred and twenty-four small neurons including 53 from control, 38 CCD and 33 CCI DRGs were recorded and analyzed. Examples of recordings and calculations of the Na^+ ^currents are shown in Fig. 2. All of the neurons analyzed and discussed in this study expressed both slow-inactivating TTX-R, and fast-inactivating TTX-S Na^+ ^currents, which we refer to as "slow" and "fast" currents, respectively. Neurons expressing predominantly (>70% of total) TTX-R currents are referred to as "slow neurons", while neurons expressing predominantly (>70% of total) TTX-S currents are referred to as "fast neurons" [6,18,19]. There were 5 controls-, 3 CCD- and 5 CCI- neurons that expressed "mixed currents" (both slow and fast currents >70% of total) were not included in the analysis of this study. The results showed that CCD treatment increased percentage of the small-slow neurons and reduced percentage of small-fast neurons, while CCI treatment decreased percentage of small-slow neurons and increased that of small-fast neurons. Data are summarized in Table 1.

**Table 1:** Proportion and distribution of the small-slow and small-fast neurons in control, CCD and CCI DRGs.

			Number of neurons (% of Total)	
			
	n	C_m _(pF)	Small-Slow	Small-Fast
			27 (51)	26 (49)
CCD	38	25.9 ± 1.2	25 (66)* #	13 (34)* #
CCI	33	24.6 ± 1.2	13 (39)*	20 (61)*

*, #, p < 0.05 indicate significant differences compared with control (*) or CCD (#) groups.

### TTX-R and TTX-S Na^+ ^current densities are reduced in small-slow neurons in CCD and CCI DRGs

**Figure 3:**
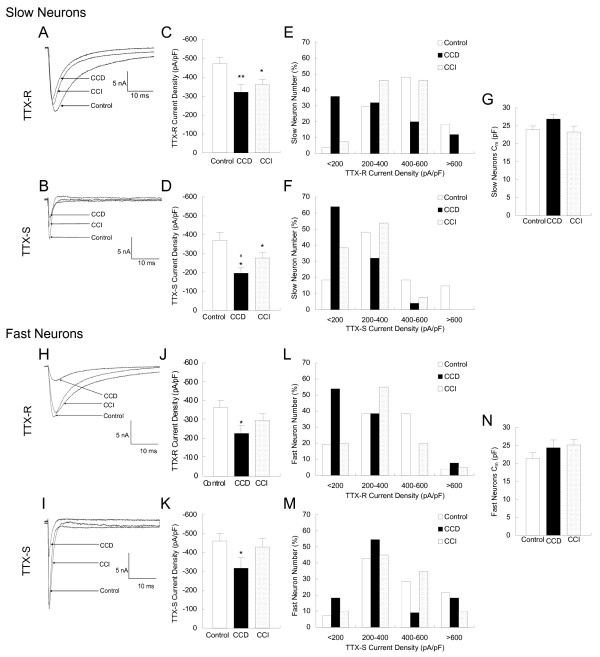
**Alteration of TTX-R and TTX-S Na^+ ^current densities in slow and fast small DRG neurons after CCD and CCI treatment.** A, B, H and I: Examples of the TTX-R and TTX-S currents recorded with the prepulse inactivation protocol with 40 ms to -10 mV test pulse in the slow neurons (A and B) and the fast neurons (H, I) from CCD, CCI and control DRGs, respectively. C, D, J and K: Alterations of the TTX-R and TTX-S current densities in the slow (C and D) and the fast (J and K) neurons. E, F, L and M: Distribution of the TTX-R and TTX-S current densities in the slow (E and F) and the fast (L and M) neurons. The densities were binned and plotted against neuron number (%). G and N: Input capacitance (C_m_) of the slow (G) and fast (N) neurons from control, CCD and CCI groups. *, p < 0.05 and **, p < 0.01 indicate significant differences compared with the control group. #, p < 0.05 indicate significant differences compared with CCI group.

The TTX-R and TTX-S current densities (peak current amplitude normalized to C_m_) were examined and compared in the small-slow neurons among CCD, CCI and control DRGs. The peak TTX-R and TTX-S current amplitudes were measured with a 40 ms test pulse to -10 mV. Examples of the TTX-R and TTX-S currents from CCD, CCI and control DRG neurons are given in Fig. 3A and 3B. CCD and CCI treatment significantly reduced TTX-R and TTX-S current densities in the small-slow neurons compared to control (Fig. 3C and 3D). The TTX-R current density was significantly reduced by approximately 30% and 20% in CCD and CCI DRGs, respectively (Fig. 3C). The TTX-S current density was reduced by approximately 50% and 25% in CCD and CCI DRGs, respectively. Reduction of TTX-S current density by CCD treatment was significantly more than that by CCI (p < 0.05) (Fig. 3D). To further demonstrate reduction in the current levels, densities were binned and plotted against neuron number (Fig. 3E and 3F). The peak distribution of TTX-R current density shifted from 400–600 pA/pF in control to the lower levels of <200 and 200–400 pA/pF in CCD and 200–600 pA/pF in CCI (Fig. 3E). The peak distribution of TTX-S current density shifted from 200–400 pA/pF in control to the levels of <200 pA/pF in CCD and <200 and 200–400 pA/pF in CCI (Fig. 3F). The C_m_, which could affect the results of the current density, was not significantly changed in CCD and CCI compared to that in control DRG neurons (Fig. 3G).

### TTX-R and TTX-S Na^+ ^current densities are reduced in small-fast neurons in CCD, but not CCI DRGs

The TTX-R and TTX-S current densities were also examined and compared in the small-fast neurons among CCD, CCI and control DRGs. Examples are given in Fig. 3H and 3I. CCD treatment significantly reduced TTX-R and TTX-S current densities approximately 40% and 30%, respectively, in the small-fast neurons compared to control (Fig. 3J and 3K). Current densities were again binned and plotted against the neuron number as shown in Fig. 3L and 3M. The peak distribution of TTX-R current densities shifted from 200–400 pA/pF in control neurons to the lower levels of <200 pA/pF in CCD (Fig. 3L). The peak distribution of TTX-S current densities shifted from 200–600 pA/pF in control neurons to the lower levels of <200–400 pA/pF in CCD (Fig. 3M). In contrast, CCI treatment failed to change the densities of both TTX-R and TTX-S currents (Fig. 3J–M). The membrane capacitance (C_m_) was not significantly changed in CCD and CCI compared to that in control DRGs (Fig. 3N).

**Figure 4:**
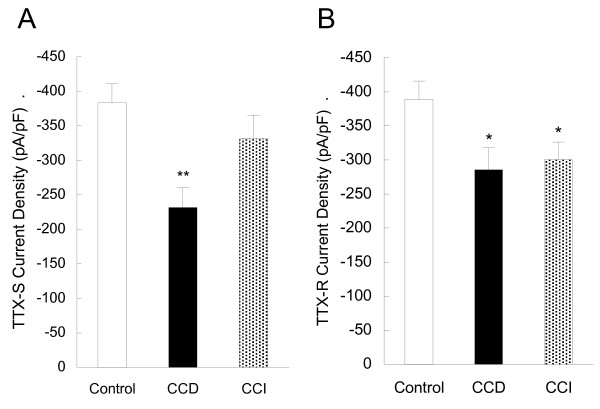
**Alteration of TTX-R and TTX-S Na^+ ^current densities in small DRG neurons after CCD and CCI treatment.** Data shown here are from Fig. 2C, D, J and 2K and the data from the slow and fast neurons are combined. CCI-induced significant alteration in TTX-S current density in the slow neurons (see Fig. 2) is hidden, while CCD-induced change in TTX-S currents still exhibit clearly (A). Both CCD- and CCI-induced reduction in TTX-R currents density is unchanged in this analysis (B).

Previous studies have shown that nerve injury including CCI do not produce significant change in the TTX-S current [16,20,21]. Here it is shown that CCI does reduce the TTX-S currents in the small-slow neurons (Fig. 3D), but not in the small-fast neurons (Fig. 3K). Interestingly, if the data from the slow and fast neurons are combined, CCI-induced alteration in TTX-S current density in the slow neurons is hidden, while CCD-induced change in TTX-S current still exhibit clearly (Fig. 4A). Both CCD- and CCI-induced reduction in TTX-R current densities are unchanged in this analysis (Fig. 4B). The C_m _was not significantly different between CCI and control DRGs and not different among the groups of CCD, CCI and control (see Table 1).

### Voltage dependence of activation of TTX-R and TTX-S Na^+ ^currents in small-slow and small-fast neurons is not altered by CCD and CCI

**Figure 5:**
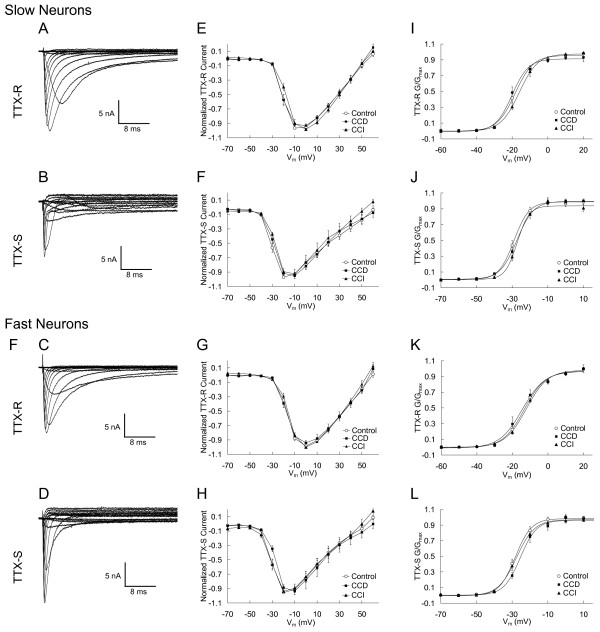
**The current-voltage relationships of TTX-R and TTX-S Na^+ ^currents obtained from either slow or fast DRG neurons are not altered by CCD and CCI treatment.** A-D: Representative currents families from the slow (A and B) and fast (C and D) neurons were recorded by using the prepulse inactivation protocol with a series of test pulse ranging from -70 mV to +60 mV (in a +10 mV increments). E-H: Normalized peak current was plotted against test pulse voltage. I-L: The conductance (G) was calculated and plotted against test pulse voltage.

Current-voltage relationship of the TTX-R and TTX-S Na^+ ^currents was measured using a I-V protocol with a 700 ms prepulse to -120 or -50 mV, followed by a series of test pulse from -70 to +60 mV with +10 mV increments. The total Na^+ ^current was obtained by using the protocol with a prepulse to -120 mV. The TTX-R component was recorded by using the protocol with a prepulse to -50 mV which inactivates TTX-S currents. The TTX-S component was obtained by digitally subtracting the TTX-R component from the total Na^+ ^current. Examples of TTX-R and TTX-S currents in the small slow and fast neurons from a control DRG are given in Fig. 5A–D. Plots of normalized peak Na^+ ^current density versus test pulse voltage for the small slow and fast neurons in control, CCD and CCI DRGs are shown in Fig. 5E–H. Activation threshold of the TTX-R currents the control neurons was between -35 and -30 mV and the maximum inward current fell between -10 and 0 mV (Fig. 5E,G). Activation threshold of the TTX-S currents in the control neurons was detected between -50 to -45 mV with maximum inward current at approximately -20 mV (Fig. 5F, H). All currents measured displayed a reversal potential (V_rev_) of about 50–55 mV, corresponding to the calculated equilibrium potential for sodium ions under these recording conditions (E_Na _= 50 mV). The voltage at which 50% of the Na^+ ^channels were activated (V_1/2_) and the slope for activation (*k*) were obtained from fitting the normalized conductance (G/G_max_)-voltage curve with the Boltzmann equation. Effects of CCD and CCI on the voltage dependence activation of TTX-R and TTX-S currents were examined and analyzed. Data are expressed and summarized in Fig. 5I–L and Table 2. In both small slow and fast neurons, neither CCD nor CCI treatment significantly altered parameters of activation of the TTX-R and TTX-S Na^+ ^currents such as V_1/2_, *k*, activation threshold, and voltage range of the maximum inward current fell in. These negative results support the findings in the current density by excluding the possibility of changes in the current amplitude caused by alternations of the activation properties of Na^+ ^currents.

**Table 2:** Voltage-dependence of activation and steady-state inactivation of the TTX-R and TTX-S Na^+ ^currents in the slow and fast small-sized control, CCD and CCI DRGs.

	TTX-R Current				TTX-S Current			
	
	V_1/2act _(mV)	*k*_act_	V_1/2inact _(mV)	*k *_inact_	V_1/2act _(mV)	*k*_act_	V_1/2inact _(mV)	*k *_inact_
Slow Neurons								
Control	-17.9 ± 1.1 (14)	4.3 ± 0.3 (14)	-35.3 ± 0.7 (14)	-4.0 ± 0.1 (14)	-26.6 ± 1.3 (14)	5.6 ± 1.1 (14)	-77.6 ± 2.2 (14)	-8.0 ± 0.5 (14)
CCD	-18.7 ± 1.7 (15)	4.9 ± 0.4 (15)	-38.8 ± 0.8** (12)	-4.6 ± 0.2* (12)	-26.2 ± 1.4 (15)	5.2 ± 0.6 (15)	-84.0 ± 1.3* (12)	-9.9 ± 0.5* (12)
CCI	-15.6 ± 1.6 (7)	5.1 ± 0.5 (7)	-38.1 ± 0.7* (7)	-4.6 ± 0.1* (7)	-26.1 ± 1.4 (7)	4.0 ± 0.8 (7)	-75.1 ± 5.1 (7)	-12.4 ± 1.8** (7)
Fast Neurons								
Control	-13.0 ± 1.2 (13)	5.6 ± 0.4 (13)	-39.4 ± 1.1 (14)	-4.4 ± 0.1 (14)	-26.2 ± 1.4 (13)	4.9 ± 0.6 (13)	-84.4 ± 1.9 (14)	-8.9 ± 0.4 (14)
CCD	-14.3 ± 1.7 (9)	5.8 ± 0.7 (9)	-37.9 ± 1.3 (7)	-5.3 ± 0.2** (7)	-23.0 ± 1.8 (9)	5.6 ± 1.1 (9)	-75.7 ± 1.5* (7)	-9.4 ± 0.8 (7)
CCI	-12.0 ± 0.8 (12)	5.9 ± 0.4 (12)	-36.8 ± 1.1 (10)	-5.0 ± 0.2* (10)	-26.8 ± 1.2 (12)	5.4 ± 1.1 (12)	-80.4 ± 2.8 (10)	-10.1 ± 0.8 (10)

V_1/2act_: membrane potential at which activation is half-maximal. *k *_act_: slope of the activation curve.

V_1/2inact_: membrane potential at which inactivation is half-maximal. *k *_inact_: slope of the inactivation curve.

The number in parenthesis indicates the number of neurons.

*, p < 0.05, **, p < 0.01 indicate significant differences compared with control group.

### Voltage-dependence of steady-state inactivation of TTX-R and TTX-S Na^+ ^currents in small-slow and small-fast neurons is altered by CCD and CCI

**Figure 6:**
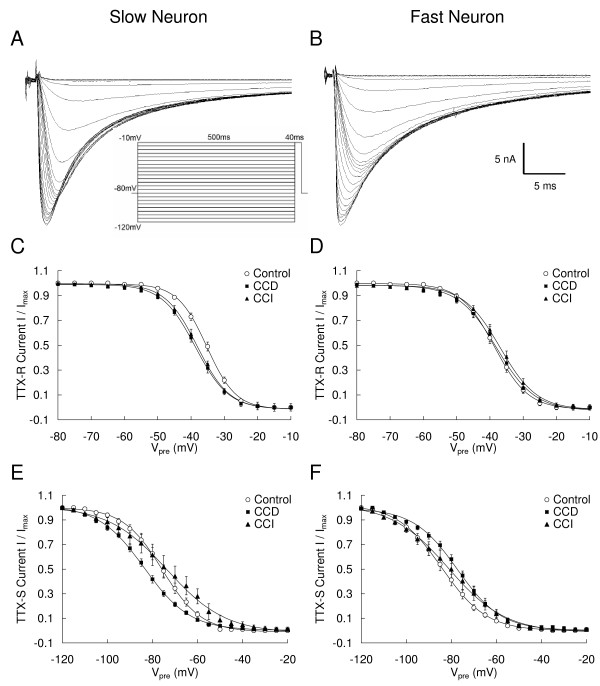
**Alteration of the steady-state inactivation of TTX-R and TTX-S Na^+ ^currents in slow and fast small DRG neurons after CCD and CCI treatment.** A and B: Representative records of the inactivation current from a slow neuron (A) and a fast neuron (B) from a control DRG. The currents were recorded by using a double protocol with a 500 ms prepulse ranging from -120 mV to -10 mV (in 5 mV increments), followed by, with a 0.8 ms interpulse interval to -80 mV, a test pulse to -10 mV. The inter-pulse period was 10 s. The TTX-R inactivation currents were measured at the time of the peak current evoked following a -50 mV prepulse. The TTX-S inactivation currents were measured at the time of the peak of the maximum current evoked following a -120 mV prepulse. Each data set was normalized and fit with a Boltzman equation. The best fitted steady-state inactivation curves were showed in C-F.

Steady-state inactivation of TTX-R and TTX-S Na^+ ^currents was measured with 500 ms prepulse to potentials over the range of -120 mV to -10 mV with 5 mV increments followed by, with a 0.8 ms interpulse interval to -80 mV, a -10 mV test pulse. The TTX-R inactivation currents were measured at the time of the peak current evoked following a -50 mV prepulse. The TTX-S inactivation currents were measured at the time of the peak of the maximum current evoked following a -120 mV prepulse [18,22]. Examples of recordings of steady-state inactivation of the TTX-R and TTX-S currents in the slow and fast neurons are shown in Fig. 6A and 6B. CCD and CCI treatment significantly altered the V_1/2 _and *k *of TTX-R and TTX-S currents in the slow and/or fast neurons as shown in Fig. 6(C–F) and summarized in Table 2. CCD and CCI produced similar effects on the TTX-R current of the slow neurons, but different on the TTX-R current of the fast neurons and the TTX-S current of the slow and fast neurons (Table 2).

**Figure 7:**
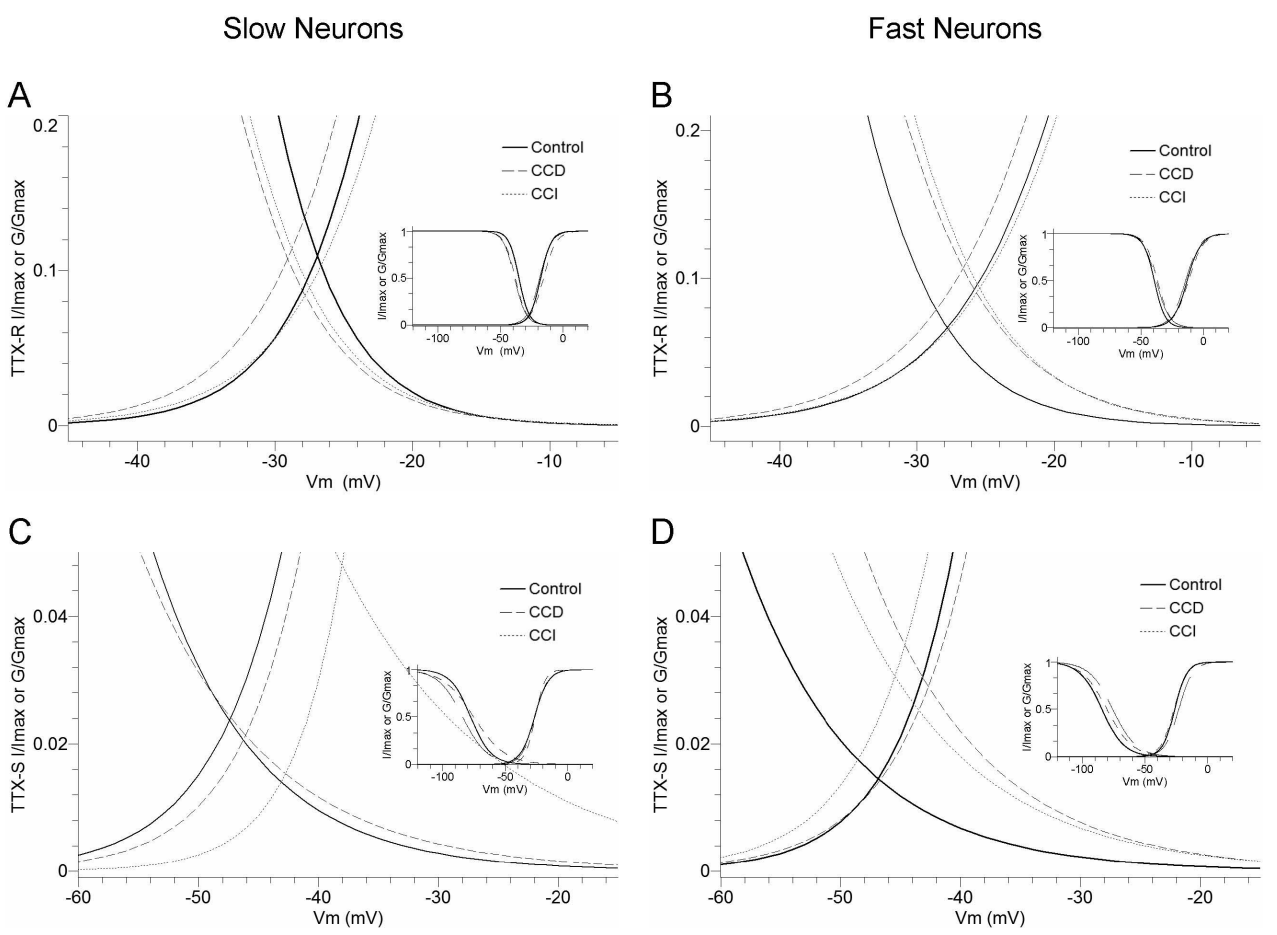
**Alteration of the window current of activation and steady-state inactivation curves in the slow and fast small DRG neurons after CCD and CCI treatment. **Boltzmann fits from the data shown in Fig. 4 and 5 are shown in A-D. The small figures inserted in each figure illustrate the overview of the activation and inactivation fit curves.

**Figure 8:**
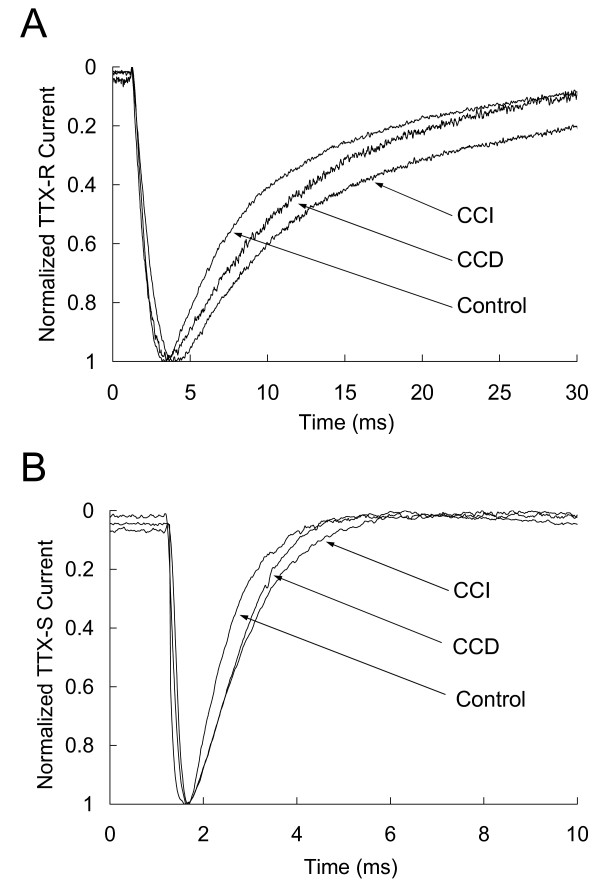
Representative recordings showing alteration of the inactivation of TTX-R (A) and TTX-S (B) Na^+ ^currents in the fast small DRG neurons after CCD and CCI treatment.

The shift in steady-state inactivation affected the window current, which is the region of overlap between the curves for the dependence of activation and inactivation. The overlapping activation/inactivation Boltzmann curves were used to determine the fraction of sodium channels activated in the peak of the window current [23,24]. Theoretical analysis of the voltage dependencies presented in Fig. 6 indicates that both CCD and CCI treatment increase the window current of the TTX-R currents in the fast, but not slow neurons (Fig. 7A and 7B). In the TTX-R currents in fast neurons at the membrane potential where maximal overlap of inactivation and activation occurred, approximately 7% of the Na^+ ^channels in control neurons were in a non-inactivated state and approximately 7% of the available channels were activated. This fraction was increased ~28% and ~56% by CCD and CCI treatment, respectively (Fig. 7B). The fractions of the window currents of the TTX-S currents were increased approximately 110% in the slow neurons and 100% in the fast neurons by CCI (Fig. 7C and 8D). In contrast, CCD treatment increased window current of the TTX-S only in the fast neurons, but not the slow neurons and the fraction was increased by ~86% (Fig. 7D).

### Inactivation of TTX-R and TTX-S Na^+ ^currents in small-fast neurons is slowed by CCD and CCI

To quantitate changes in decay rate of the TTX-R and TTX-S Na^+ ^currents, we fit the currents with single exponentials as that previously described [25]. The results showed that the inactivation of TTX-R currents in the small-fast neurons was significantly slowed by CCI treatment from 6.09 ± 0.48 ms in control to 8.34 ± 1.12 ms (p < 0.05), but not by CCD treatment although the inactivation tended to be slower (p > 0.05) (Fig. 8A, Table 3). In contrast, inactivation of TTX-S Na^+ ^currents in the fast neurons was significantly slowed by both CCD and CCI treatment (Fig. 8B, Table 3). Neither CCD nor CCI treatment significantly altered inactivation of both TTX-R and TTX-S currents in the slow neurons (Table 3).

**Table 3:** Inactivation of the TTX-R and TTX-S Na^+ ^currents of small-slow and small-fast neurons evoked with -10 mV test pulse in control, CCD and CCI DRGs.

	n	Current inactivation time constant (ms)	
		
		TTX-R	TTX-S
Small-Slow Neurons			
Control	27	5.11 ± 0.42	0.90 ± 0.07
CCD	25	4.82 ± 0.29	1.00 ± 0.08
CCI	13	6.65 ± 0.89	1.10 ± 0.12
Small-Fast Neurons			
Control	26	6.09 ± 0.48	0.92 ± 0.05
CCD	13	7.18 ± 0.54	1.12 ± 0.09*
CCI	20	8.43 ± 1.12*	1.17 ± 0.07**

*, p < 0.05, **, p < 0.01 indicate significant differences compared with control group.

## Discussion

The present study investigated alterations of TTX-R and TTX-S Na^+ ^currents in the small DRG neurons after DRG somata compression (CCD treatment) and compared the different effects of DRG somata compression and the axons injury (CCI treatment) on the Na^+ ^currents. The principle findings are 1) CCD treatment significantly reduces the TTX-R and TTX-S current densities in the small-slow and small-fast subtypes of DRG neurons; 2) CCD alters voltage-dependent steady-state inactivation of the TTX-R and TTX-S currents and increases window current of the activation and inactivation, but exhibits different effects on V_1/2 _and *k *of the TTX-S currents in both slow and fast neurons; 3) CCD reduces the decay rates of the TTX-S, but not TTX-R currents inactivation in the fast neurons; and 4) CCI shows different effects from CCD in most of the tested properties of the TTX-R and TTX-S currents. These findings suggest a possible sodium channel mechanism underlying CCD-induced DRG neuron hyperexcitability and indicate that injuries to DRG somata and peripheral axons may result in different alterations of the VGSCs.

The DRG neurons, particularly the nociceptive small neurons, are notable in expressing multiple sodium channel isoforms including Nav1.8 and Nav1.9 contributing to the TTX-R currents, and Nav1.7, Nav1.6, Nav1.5, Nav1.3, Nav1.2 and Nav1.1 contributing to the TTX-S currents [26-31]. Many of these sodium channels can be dynamically regulated after nerve injury and/or inflammation and the specific channels may play crucial roles in nociception. Several lines of studies including many with transgenic mice lines have clearly implicated Nav1.7, Nav1.8 and Nav1.9 in inflammatory and probably neuropathic pain [1,2]. The present study shows that CCD treatment significantly down-regulates both the TTX-R and TTX-S Na^+^currents in the small DRG neurons. The TTX-R Na^+ ^currents recorded in our study are predominantly the Nav1.8 as identified by the specific protocol [1]. Such alteration of TTX-R Na^+^currents after CCD treatment is consistent to majority of the previous findings that Nav1.8 mRNA, protein and current are substantially decreased in DRG neurons in axotomized DRG neurons [6-9] or sciatic nerve injury [10,16]. These observations demonstrate a common role for the TTX-R Na^+ ^channels in the DRG neurons after injury to either somata or peripheral axons. A recent study indicates that the cutaneous, medium-sized dissociated CCD DRG neurons exhibit an increase in TTX-R Na^+ ^currents [15]. Such different changes of Na^+ ^currents in different types of DRG neurons were also found in rats that received CCI treatment [16], indicating that TTX-R Na^+ ^currents may have different functions in the small- vs the medium-sized DRG neurons after neuron injury, and may contribute to the different mechanisms of these neurons in the abnormal neuron excitability. TTX-R Na^+ ^channel is also found upregulated in the sciatic nerve axons at the site of injury [32] and redistributed in the uninjured adjacent axons of DRG neurons [33]. Consistently, contribution of Nav1.8 currents to neuropathic pain conditions has been demonstrated controversial. Another interesting finding in the present study is that CCI treatment reduces the TTX-R currents only in the slow, but not the fast neurons, further supporting that nerve injury can produce different effects on sodium channels in different types of DRG neurons. In addition, the Nav1.9 channel can have profound effects on resting membrane potential (RMP), and thus on excitability in DRG neurons [26,27]. Nav1.9 current activates at more negative potentials (-80 mV), differing from the Nav1.8 current activating at potentials close to RMP (-60 to -70 mV), and generates the persistent TTX-R current identified [26,34,35]. Nav1.9 current plays an important role in setting RMP as well as contributing to subthreshold electrogenesis in small DRG neurons [26,27]. Nav1.9 was not observed in the present study because of ultraslow inactivation at the holding potentials used and at the time domain of the pre-pulse 700 ms to -120 mV applied to remove the fast inactivation of TTX-S currents. This yielded an estimation of the sodium current in the cell minus the Nav1.9 current.

The TTX-S Nav1.7 and Nav1.3 channels have been identified to play important roles in neural hyperexcitability and chronic pain [1], but the observations again are controversial. Nav1.7, Nav1.6 and Nav1.3 channels are upregulated [36-39] or down-regulated [25,39-41] in DRG neurons after nerve injury or axotomy. In the present study, CCD and CCI treatment both result in down-regulation of TTX-S currents. However, CCI treatment selectively reduces density of TTX-S currents in the slow, but not the fast neurons. These results suggest different roles for TTX-S currents in these two subtypes of neurons after peripheral nerve injury. This differentiation might in some way link to the conflict findings in the experiments that knock-down Nav1.3 leads to decrease [42] or no change [43] in pain sensitivity. The differential effects in the TTX-R and TTX-S Na+ current induced by CCD and CCI may contribute partly to certain differences in neural excitability and behavioral manifestations between the two models [13]. In addition, it is worthy while to mention that because only a proportion of the L4/5 DRG neurons were directly injured in the CCI model (due to the contribution of L4/5 to sciatic nerve in the injured level), the neurons under investigation might also include some intact uninjured ones [7] and therefore the sensitivity of statistics could be lowered.

How these differential alterations of sodium channels/currents would contribute to neural excitability and neuropathic pain remains unclear [1,2]. A recent study by Rush et al [44] may provide an explanation to such controversial and conflict observations. It is shown that a single sodium channel (Nav1.7) mutation can produce opposing phenotypes (hyperexcitability vs hypoexcitability) in sensory neurons and sympathetic neurons, respectively, and the selective presence of the Nav1.8 channel is a major determinant of these opposing effects. Majority of nociceptive DRG neurons express Nav1.8 [29,45], which contributes most of the current underlying the action potential upstroke [9,46]. Because it has depolarized voltage-dependence of activation and inactivation [6,10,45,47] compared with other sodium channels, Nav1.8 permits DRG neurons to generate action potentials sustain repetitive firing when depolarized [9,46]. This finding suggests that the physiological coupling of Nav1.8 and Nav1.7 in the nociceptive DRG neurons may contribute to the phenotypes in the different cell types as well as in the different neuropathic conditions. In the same study [44], RMP of the sensory and sympathetic neurons is depolarized following Nav1.7 mutation and such depolarization is thought to be a result of increased window currents [23,44]. Interestingly, a similar depolarization is also true in the large- and medium-sized and small DRG neurons after CCD treatment as we demonstrated recently [14] and the window currents are increased in the small-fast neurons after CCD treatment and in the small-slow neurons after CCI treatment, while the density of both TTX-R and TTX-S Na+ currents is downregulated, as shown in the present study. These findings might support a possibility that either upregulation or downregulation of the TTX-R or TTX-S current densities in the injured medium-sized [15,8] and small DRG neurons, which express both Nav1.8 and Nav1.7 channels, can produce neural hyperexcitability. Such alteration of Na+ channels/currents on neural function should also depend on the basis of the cell background in which the alteration is expressed. Further investigation and analysis are urgently needed to elucidate such complex relationships between neural hyperexcitability and alterations of the sodium channels after nerve injury.

Steady-state inactivation of TTX-R and TTX-S Na^+ ^currents alters and exhibits different changes in the midpoints (V_1/2_) and slopes (*k*) of inactivation curves in the small-slow and small-fast neurons after CCD and CCI treatment. It is interesting that both CCD and CCI treatment increase the window current in both slow and fast neurons by differently (depolarizing or hyperpolarizing) shifting the steady-state inactivation curves midpoint and decreasing the slope factor. The window current (overlap between the curves for the dependence of activation and inactivation) represents a voltage region in which sodium channels can continue to open because some channels are activated and not all of the channels are inactivated. The increased window current therefore may result in an increase in the persistent current that can seize activity and may affect neuronal excitability [23,24]. These alternations of steady-state inactivation curves therefore show a potential to depolarize the resting membrane potential and increase the neuronal excitability. A recent study indicates that the CCD treatment hyperpolarizing shifts the activation curves of the TTX-S current, but not the steady-state inactivation curves in cutaneous medium-sized DRG neurons [15]. Such a hyperpolarizing shift of the activation curve may also increase the window current. Thus, the increased window current may underlie the neural hyperexcitability. In addition, our results show that inactivation of the TTX-R and TTX-S currents are slowed down by CCD and/or CCI in the small-fast neurons. This may also contribute to the neural hyperexcitability. We hypothesize that alterations of the sodium channel gating properties associated with down- or up-regulation of the current density may contribute to the neural hyperexcitability, while redistribution of the sodium channels to the adjacent uninjured fibers may contribute to the development of neuropathic pain [33]. This hypothesis may also provide an explanation for the controversial observations.

CCD treatment produces local inflammation particularly in the first postoperative week as described in the previous studies [12,13,48], in addition to producing compression of the ganglion. In this study, the down-regulation of the TTX-R currents are similar to those observed in the nerve injury model, but not the inflammation model in which up-regulated [1]. In the present study, the DRG neurons were isolated from rats 10–14 days after continuous compression. We noticed that the inflammation during this period of time was much less than that in the first week after the rod was initially introduced into the intervertebral foramen (data not collected), which was described in our previous studies [12,13,48]. Such a point may also contribute to the difference between our study and Tan et al [15], in addition to the different types of DRG neurons recorded.

It needs to be pointed out that nerve injury alters the electrophysiological properties of diverse types of primary afferent neurons and triggers a myriad of changes in gene expression that affect many proteins, including ion channels, receptors, and other membrane proteins [49-52]. Such alterations are likely to complicate the changes in Na^+ ^currents we observed. These complexities might be reduced by sampling functionally homogeneous subpopulations or recording from the same neurons before and after injury. While this can be done with some invertebrate nociceptors [53,54], it is not yet practical for DRG neurons.

Additional ion channel mechanisms may also contribute to neural hyperexcitability and behavioral hyperalgesia and allodynia after DRG compression. Recent studies have shown that CCD causes a decrease in fast-inactivating K^+ ^current [15] and an increase in expression of a hyperpolarization-activated cation current (*I*_*h*_), in addition to an increase in TTX-R Na^+ ^currents in cutaneous, medium-sized DRG neurons [55]. The *I*_*h *_current is activated during the afterhyperpolarization that follows an action potential and leads to a sustained depolarizing current, resulting in repetitive firing [55].

## Conclusion

In summary, this study shows that CCD treatment can cause profound changes in densities and properties of inactivation of TTX-R and TTX-S Na^+ ^currents of the small DRG neurons, and that DRG somata compression results in different alterations of the Na^+ ^currents from the peripheral nerve injury. The findings also point to a complexity of hyperexcitability mechanisms contributing to CCD and CCI hyperexcitability in small DRG neurons,

## Methods

### Animals and surgical procedures

Experiments were performed on adult, male Sprague-Dawley rats (n = 32, 200–250 g). The rats were housed in groups of 3–4 in plastic cages (40 × 60 × 30 cm) with soft bedding and free access to food and water under a 12-h day/12-h night cycle. Under these conditions, they were kept 3–5 days, before and up to 14 days after surgery and/or treatment. The animals were divided into groups as described below (CCD, CCI and Control). All surgeries were done under anesthesia induced by intraperitoneal injection (i.p.) of sodium pentobarbital (40 mg/kg). After surgery, the muscle and skin layers were sutured. These procedures were conducted in agreement with the regulations of the ethics committee of the International Association for the Study of Pain, the National Institute of Health guide for the care and use of Laboratory animals and approved by Parker Research Institute Animal Care and Use Committee.

DRG compression was produced by surgically implanting stainless steel rods unilaterally into the intervertebral foramen at L_4 _and L_5 _using the procedure for CCD we previously described [12,13]. In brief, the rats (n = 12) were anesthetized; paraspinal muscles were separated from the mammillary and transverse processes, and the intervertebral foramina of L_4 _and L_5 _were exposed. One stainless steel L-shaped rod, 4 × 2 mm in length and 0.6 mm in diameter, was implanted into the foramen at L_4 _and another at L_5_. CCI model was employed to produce peripheral nerve injury and the procedure was similar to that described in the CCI model [56]. The left common sciatic nerve of each rat (n = 10) was exposed at the level of mid-thigh. Proximal to the sciatic nerve's trifurcation, about 7 mm of nerve was freed of adhering tissue and four ligatures (4-0 chronic gut) were tied loosely around it with about 1 mm spacing. The length of affected nerve was about 5 mm. Another group of rats (n = 10) received neither surgery nor injury and were served as control. A series of previous studies in our lab and others has shown that sham surgery for CCD and CCI treatment did not produce significant electrophysiological differences between neurons from previously unoperated versus sham-operated controls [13-15,21], therefore, the sham operations were not considered necessary in the present study.

### Behavioral testing

Thermal hyperalgesia was indicated by a decrease in the latency of foot withdrawal evoked by a radiant heat stimulus as described previously [13,14]. The IITC Model 336 Analgesia Meter (Life Science, Series 8) providing a heat source was used in the present study. In brief, each rat was placed in a box (22 × 12 × 12 cm) containing a temperature-controlled smooth glass floor associated with the Analgesia Meter. The heat source was focused on a portion of the hindpaw, which was flush against the glass, and delivered until the hindpaw moved or up to 20 sec to prevent tissue damage. The range of latency of foot withdrawal in naïve, control rats was 9–15 sec. Thermal stimuli were delivered 4 times to each hind paw at 5–6 min intervals. The rats were tested on each of 2 successive days prior to surgery (the first test was at 2 days and the second at 2 hours prior to surgery). Postoperative tests were conducted on the day of electrophysiological recording (days 10–14). Thermal hyperalgesia for a given rat was defined as a postoperative decrease of foot withdrawal latency from the mean preoperative value, with a difference score ≥ 3 s [14]. Only rats that exhibited thermal hyperalgesia after CCD or CCI treatment were used for the electrophysiological studies.

### Dissociation of DRG neurons

DRG neurons were dissociated from L_4 _and/or L_5 _ganglia taken from 8 CCD, 8 CCI and 8 Control rats. The protocol was the same as that we have described recently [57]. In brief, the excised ganglion was minced using microdissection scissors, the DRG fragments transferred into 10 ml of the buffered solution containing collagenase (type IA, 1 mg/ml, Sigma) and trypsin (0.5 mg/ml, Sigma), and then incubated for 30 min at 35°C. The DRG fragments were removed, rinsed 2–3 times in the buffered solution, and put into the buffered solution (5 ml) containing DNase (0.2 mg/ml, Sigma) to prevent possible toxicity from DNA leaking from ruptured cells. Individual neurons were dissociated by passing DRG fragments through a set of fire-polished glass pipettes with decreasing diameter.

### Voltage-clamp electrophysiology

Voltage-clamp recordings were performed in the dissociated small DRG neurons with the standard whole-cell patch-clamp configuration. All recordings were conducted at room temperature (20~22°C) and during 2~8 hrs after dissociation. Fire-polished electrodes were fabricated from 1.5 mm out diameter borosilicate capillary glass (Sutter Instruments, Novato, CA) by using a Sutter P-97 puller (Sutter Instruments, Novato, CA), and had a resistance of 1 – 3 MΩ. The pipette solution contained (in mM): 110 CsF, 11 EGTA, 10 NaCl, MgCl_2 _5, and 10 HEPES, pH 7.3 with CsOH. Isolated sodium current was recorded from the single neuron in the presence of a bath solution that contained (in mM): 65 NaCl, 2.5 KCl, 5 MgCl_2_, 0.01 CaCl_2_, 50 Choline-Cl, 20 TEA-Cl, 5 glucose, 5 Na-HEPES, and 5 HEPES, pH 7.4 with NaOH. Bath solution was applied to the recording chamber and removed via a Peri-Star Pro peristaltic pump (World Precision Instruments, Sarasota, FL).

Voltage-clamped currents were recorded with an Axopatch-200B amplifier (Molecular Devices, Union city, CA). Data were acquired on a PC computer with the Clampex v10.0 software (Molecular Devices), filtered with a low-pass Bessel filter setting of 5 kHz and digitized at a sampling rate of 40 kHz via a Digidata 1440A analog-to-digital converter (Molecular Devices). The membrane capacitance (C_m_) was read from the amplifier by software Clampex v10.0 for determining the size of cells and calculating the current density. Voltage errors were minimized by using 80–90% series resistance compensation and the capacitance artifact was canceled by the patch-clamp amplifier. Linear leakage currents were digitally subtracted on-line using hyperpolarizing potential after the test pulse (P/6 procedure). Data acquisition began 5 min after establishing whole-cell configuration and the holding potential was at -80 mV.

Somata of the small DRG neurons were classified by their diameters (15 ~30 μm) and C_m _(≤ 45 pF). Neurons were not considered for analysis if they had high leakage currents (holding current >1.0 nA at -80 mV), membrane blebs, total sodium current < 500 pA, or an access resistance > 5 MΩ. Access resistance was monitored throughout the experiment and data were not used if resistance changes of >20% occurred. Data were not corrected to account for liquid junction potential. The offset potential was zeroed before patching the cells and checked after each recording for drift.

To analyze the voltage dependence of channel activation, the sodium conductance (G) was calculated. Peak current data for each cell were divided by the respective driving force (V_m _- V_rev_), plotted against V_m_, and fit to a Boltzmann distribution equation of the following form:

G = G_max_/(1 + exp((V_1/2 _- V_m_)/*k*)),

Where G_max _is the maximum G, V_1/2 _is the potential at which activation is half-maximal, and *k *is the slope of the curve.

For the analysis of steady-state inactivation kinetics, the inactivation parameter was fitted to a Boltzmann distribution equation:

I/I_max _= 1/(1 + exp ((V_1/2 _- V_pre_)/*k*)),

Where I_max _is the maximum sodium current elicited after the most hyperpolarized prepulse, the V_pre _is the prepulse potential, V_1/2 _is the potential at which inactivation is half-maximal, and *k *is the slope factor.

### Statistical tests

The student *t*-test was used to examine the differences in mean latency of thermal paw withdrawal between preoperative (mean value of the two preoperative tests) and postoperative on the day of electrophysiological recordings. The specific hypotheses about differences between each treated (CCD or CCI) and the control group for each electrophysiological parameter was examined. Comparisons among CCD, CCI and control groups were performed with one-way ANOVA followed by Newman-Keuls tests. *X*^2 ^tests were used to identify differences in the incidence of effects. All data are presented as mean ± SE. Statistical results are considered significant if *p *< 0.05.

## Abbreviations

CCD: Chronic compression of dorsal root ganglion; CCI: Chronic constriction injury of the sciatic nerve; DRG: Dorsal root ganglion; TTX-R: Tetrodotoxin-resistant; TTX-S: Tetrodotoxin-sensitive; VGSCs: Voltage-gated sodium channels

## Competing interests

The authors declare that they have no competing interests.

## Authors' contributions

XJS and ZJH planned the studies. ZJH conducted the experiments, analyzed the data and contributed to the writing of the paper. XJS participated in the studies and data analysis and wrote the paper. Both authors approved the final manuscript.
